# RSV Vaccine Based on Rhabdoviral Vector Protects after Single Immunization

**DOI:** 10.3390/vaccines7030059

**Published:** 2019-07-03

**Authors:** Sarah Wilmschen, Sabrina Schneider, Felix Peters, Lea Bayer, Leila Issmail, Zoltán Bánki, Thomas Grunwald, Dorothee von Laer, Janine Kimpel

**Affiliations:** 1 Division of Virology, Medical University of Innsbruck, 6020 Innsbruck, Austria; 2 Department of Immunology, Fraunhofer Institute for Cell Therapy and Immunology IZI, 04103 Leipzig, Germany

**Keywords:** VSV-GP, viral vector vaccine, respiratory syncytial virus (RSV), neutralizing antibodies

## Abstract

The respiratory syncytial virus (RSV) is one major cause of lower respiratory tract infections in childhood and an effective vaccine is still not available. We previously described a new rhabdoviral vector vaccine, VSV-GP, a variant of the vesicular stomatitis virus (VSV), where the VSV glycoprotein G is exchanged by the glycoprotein GP of the lymphocytic choriomeningitis virus. Here, we evaluated VSV-GP as vaccine vector for RSV with the aim to induce RSV neutralizing antibodies. Wild-type F (F_wt_) or a codon optimized version (F_syn_) were introduced at position 5 into the VSV-GP genome. Both F versions were efficiently expressed in VSV-GP-F infected cells and incorporated into VSV-GP particles. In mice, high titers of RSV neutralizing antibodies were induced already after prime and subsequently boosted by a second immunization. After challenge with RSV, viral loads in the lungs of immunized mice were reduced by 2–3 logs with no signs of an enhanced disease induced by the vaccination. Even a single intranasal immunization significantly reduced viral load by a factor of more than 100-fold. RSV neutralizing antibodies were long lasting and mice were still protected when challenged 20 weeks after the boost. Therefore, VSV-GP is a promising candidate for an effective RSV vaccine.

## 1. Introduction

Respiratory syncytial virus (RSV), first isolated in 1955, belongs to the family *Paramyxoviridae* and is classified in two antigenic subgroups, A and B [1]. RSV has a single-stranded, non-segmented (-)sense RNA genome of ~15 kb which encodes 11 genes [2,3]. RSV is one major cause of lower respiratory tract infections in childhood. According to the World Health Organization (WHO), about 33 million new infections are registered worldwide every year and more than 3 million infected individuals develop severe symptoms that require hospitalization [4]. Until now, Palivizumab—a humanized monoclonal antibody directed against the RSV fusion (F) glycoprotein—is the only prophylactic treatment for high-risk infants. When injected monthly at a dose of 15 mg/kg, the rate of hospitalization is reduced by 55%, indicating that neutralizing antibodies (nAbs) can prevent severe diseases caused by RSV infections [5,6]. However, due to high costs, Palivizumab is recommended only for high risk infants and a vaccine is still needed. The titer of RSV-specific (neutralizing) Abs is ‘the correlate of protection’ (CoP), while immune responses from CD4^+^, CD8^+^ and innate cells are of minor importance and termed ‘co-CoPs’ [7]. High titers of RSV nAbs reduce the risk of hospitalization [8]. Therefore, RSV vaccine candidates usually aim to induce high titers of RSV nAbs either in the primary target population of RSV, infants and elderly, or in pregnant women to transfer the antibodies to the child. It was shown in mice, cotton rats and humans that nAbs can be transplacentally transferred from mother to the offspring and confer protection from challenge with RSV [9,10,11,12].

The RSV glycoprotein G and the fusion protein F are the two major surface glycoproteins on the RSV virion and therefore the major targets for nAbs induction. RSV G binds to heparin sulfate and thereby mediates attachment of the virion to the target cell [13,14]. Once attached to the target cell, RSV F protein mediates the fusion of the viral with the target cell membrane. However, a ΔG RSV variant is infectious on some cell lines as for example Vero cells, showing that under certain conditions F can mediate both functions; attachment and fusion [15]. On the surface of the virion, F protein is present as a functional trimer in a pre-fusion conformation [16]. Prior to membrane fusion, RSV F undergoes conformational rearrangement from the metastable pre-to the stable postfusion structure upon a—yet unknown—trigger [17]. Neutralizing and protective responses against the G protein are serotype-specific while antibodies against F are broadly cross-reactive between both RSV subgroups and highly neutralizing [17,18]. As compared to the G protein, RSV F has high sequence conservation (amino acid sequence identity between the two RSV subgroups of 53% and 89%, for G and F respectively) [1,19]. Hence, RSV F is an ideal vaccine antigen to induce potent, cross-reactive RSV nAbs.

In a first attempt to generate a protective vaccine for RSV, a formalin inactivated RSV (FI-RSV) was used to vaccinate antigen-naïve infants. Although FI-RSV induced high levels of RSV F-specific antibody levels in serum, vaccinated infants unexpectedly developed more severe illness after natural infection, compared to the control group of infants receiving a similar parainfluenza type 1 vaccine; 80% of the vaccinated infants required hospitalization, in contrast to 5% in the control group, and two FI-RSV vaccinated children died [20,21,22]. Subsequent studies have shown that the enhanced disease after immunization with FI-RSV can be attributed to a skewed type 2 helper (Th2) response of CD4^+^ T cells [23,24,25,26]. Additionally, FI-RSV induced antibody responses with only weak neutralizing activity, probably due to the denaturation of the antigen [27,28]. In a very recent study, it was shown that RSV inactivation by formalin produced a highly divergent degree of RSV F antigenicity, which might explain the different outcomes seen in several preclinical trials [29].

Therefore, in the case of RSV, viral vectors which present intact RSV F in the native conformation are a promising alternative. VSV-GP, a variant of the vesicular stomatitis virus (VSV), where the glycoprotein G of VSV is exchanged by the glycoprotein GP of the lymphocytic choriomeningitis virus (LCMV), is a potent candidate vaccine vector [30,31]. We previously showed that VSV-GP overcomes the two major limitations of the parental VSV wild type, namely its neurotoxicity and the rapid induction of vector neutralizing antibodies already after the first application [30,32]. On the other hand, VSV-GP induces potent T cell and antibody responses against virus-encoded vaccine transgenes [30].

In the current study, we evaluated VSV-GP as vaccine vector for RSV. Upon immunization with VSV-GP expressing full length, codon-optimized RSV F (VSV-GP-F_syn_), mice produced high titers of RSV neutralizing antibodies, which could be boosted once. In a subsequent challenge with a pathogenic RSV strain, mice were protected with a ~2–3 log reduction in viral load in the lung. This protection was already seen after a single intranasal immunization. Protection, as well as nAb titers, were long lasting since the reduction of viral load was still seen when animals were challenged 20 weeks after boost. These results show that VSV-GP-F_syn_ is a highly potent vaccine candidate against RSV. In addition, VSV-GP as replication competent viral vector is a versatile vaccine platform for potentially many further indications.

## 2. Materials and Methods

### 2.1. Ethic Statement

Animal experiments were performed in compliance with the national animal experimentation law (“Tierversuchsgesetz”) and animal trial permission was granted by national authorities (Bundesministerium für Wissenschaft und Forschung, # BMWFW-66.011/0139-WF/V/3b/2014 and BMWFW-66.011/0036-V/3b/2018).

### 2.2. Cell Lines

BHK-21 cells (American Type Culture Collection, Manassas, VA, USA) were maintained in Glasgow minimum essential medium (GMEM) (Gibco, Carlsbad, CA, USA) supplemented with 10% fetal calf serum (FCS, Thermo Fisher Scientific, Vienna, Austria), 5% tryptose phosphate broth (Gibco, Carlsbad, CA, USA), 100 units/mL penicillin (Gibco, Carlsbad, CA, USA), and 0.1 mg/mL streptomycin (Gibco, Carlsbad, CA, USA). 293T (ATCC^®^ CRL-3216™) and Vero cells (ATCC^®^ CCL81™) were cultured in Dulbecco’s Modified Eagle‘s Medium (DMEM, Lonza Walkerville, MD, USA) supplemented with 10% FCS, 2 mM L-glutamine, 100 units/mL penicillin and 0.1 mg/mL streptomycin. Human epithelial cells type 2 (HEp2) were maintained in Dulbecco’s modified Eagle’s medium (DMEM, Lonza Walkerville, MD, USA) with GlutaMAX (Thermo Fisher Scientific, Vienna, Austria), containing 10% FBS and Penicillin/Streptomycin (Thermo Fisher Scientific, Vienna, Austria) at 37 °C with 5% CO_2_.

### 2.3. Viruses

VSV, VSV*ΔG (recombinant VSV Indiana strain lacking the viral envelope protein G) and VSV-GP have been described previously [32]. VSV-GP variants containing either the wild type RSV fusion protein (F) or a codon optimized variant of RSV F (GenBank entry: EF566942) were generated by replacing luciferase with the corresponding RSV F variant in VSV-GP-Luciferase, a VSV-GP variant containing luciferase as additional transgene on position 5 [33]. Newly generated VSV-GP variants were rescued as previously described and subsequently twice plaque purified. All VSV and VSV-GP variants were produced on BHK-21 cells and concentrated via low speed overnight centrifugation through a sucrose cushion. Stocks were titrated on BHK-21 cells via tissue culture infectious dose 50% (TCID_50_) assay. RSV and rgRSV, an RSV variant containing GFP as additional transgene, was kindly provided by M. Peeples and P. Collins (NIH, Bethesda, MD, USA). Stocks were produced and titrated as described previously [34].

FI-RSV was prepared according to the protocol for “Lot100” [20]. Briefly, RSV-containing cell culture supernatant was incubated with a final concentration of 0.025% (*v/v*) formaldehyde (F1635-25ML, Sigma-Aldrich) for 96 h at 37 °C. The virus was then precipitated using aluminum hydroxide.

### 2.4. Replication Kinetics

BHK-21 or Vero cells were infected with an multiplicity of infection (MOI) of 0.05 of VSV-GP variants in triplicates. One hour after infection, the inoculum was removed, cells were washed twice with phosphate-buffered saline (PBS) and fresh medium was added. At indicated time points, supernatant was collected, centrifuged to remove cell debris and stored at −80 °C. Viral titers were determined via TCID_50_ assay.

### 2.5. TCID_50_ Assay

Titers of VSV-GP variants were determined via TCID_50_ assay using the method of Spearman–Kärber as described previously [35]. Ten-fold serial dilutions of virus samples were added in eight replicates to BHK-21 cells. Six days after infection, the number of living and of dead wells was counted for each dilution and the titer was calculated.

### 2.6. In Vitro Infection for Western Blotting

BHK-21 cells were infected with an MOI of 0.1 and 0.01 of VSV-GP variants. One hour after infection, the inoculum was removed, cells were washed twice with PBS and fresh medium was added. One day after infection, cell lysates were prepared and stored at −80 °C. Samples were analyzed by Western blotting.

### 2.7. Western Blotting

Cell lysates were prepared using ice-cold cell-lysis buffer (50 mmol/L HEPES, pH 7.5; 150 mmol/L NaCl; 1% triton X-100; 2% aprotinin; 2 mmol/L ethylenediaminetetraacetic acid (EDTA), pH 8.0; 50 mmol/L sodium fluoride; 10 mmol/L sodium pyrophosphate; 10% glycerol; 1 mmol/L sodium vanadate; and 2 mmol/L Pefabloc SC) for 30 min. Subsequently, samples were centrifuged at 13,000 rpm for 10 min in a table top centrifuge. Supernatants were transferred to new tubes and stored at −80 °C till use. A total of 10 µL of virus sample was mixed with loading dye. SDS-PAGE of proteins was performed on a 10% polyacrylamide gel (for detection of RSV F and VSV N: heated loading buffer without β-Mercaptoethanol added to samples; for detection of LCMV GP: samples boiled in loading buffer with β-Mercaptoethanol). Subsequently, proteins were transferred to 0.45 µm nitrocellulose membranes (Whatman, Dassel, Germany) and membranes were blocked with MPBST (PBS containing 5% skim milk and 0.1% Tween-20). Proteins were detected using primary antibodies (RSV F (18F12) [36], VSV N (Kerafast, Boston, USA), β-actin (clone AC-74, Sigma-Aldrich, St. Louis, MO, USA)) or hybridoma supernatant (LCMV GP (KL25)) and appropriate peroxidase-conjugated secondary antibodies.

### 2.8. Production of LCMV GP and/or RSV F Pseudotyped Single-Cycle Infectious VSV*ΔG Viruses

First, 10 cm dishes with 239T cells were transfected by calcium-phosphate method with expression plasmids for the different RSV F variants (10 µg per dish). In half of the dishes, 2 µg of an LCMV GP expression plasmid were added. Cells were co-transfected with 1 µg of an human immunodeficiency virus (HIV) Rev expression plasmid and 2 µg of a GFP expression plasmid to monitor transfection efficacy using fluorescent microscopy. After 24 h, successful transfection was assessed by microscopic analysis of GFP^+^ cells and cells were either harvested for Western blot analysis or infected with an MOI of 0.5 of a VSV*ΔG virus that had been produced on VSV G expressing cells. Two hours after infection, the inoculum was removed, cells were washed with PBS and fresh medium was added. One day after infection, supernatant was harvested, 0.45 µm sterile filtered and virus was concentrated via low speed overnight centrifugation through a sucrose cushion. Virus pellets were resuspended in PBS and stored at −80 °C.

Infectivity of RSV F and/or LCMV GP pseudotyped VSV*ΔG particles was assessed via flow cytometry. First, the newly produced virus stocks were incubated for 1 h at 4 °C with a mouse serum containing neutralizing antibodies against VSV G to neutralize any remaining infectivity mediated by G from the production virus. Subsequently, Vero cells were infected with the viruses. Two hours after infection, the inoculum was removed, cells were washed with PBS and fresh medium was added. One day after infection, cells were trypsinized, washed twice with FACS-Buffer and GFP^+^ cells were quantified by FACS (BD FACSCanto™ II system). Virus particles/mL were calculated with the following formula: (Cell number × % GFP^+^ cells × dilution factor) ÷ 100 = Virus particles/mL

### 2.9. Analysis of Viral Particles by FACS

First, 5 × 10^7^ TCID_50_ of VSV, VSV-GP, VSV-GP-F_wt_ or VSV-GP-F_syn_ were coupled to Adju-Phos^®^ (a kind gift by Brenntag Biosector A/S, Ballerup, Denmark, 10 µL of a 1:100 dilution per sample) for 30 min at 37 °C. PBS was added, samples were centrifuged for 5 min at 2,000 rpm and supernatant removed with a vacuum pump. Adju-Phos^®^ particles with bound virus were blocked for 30 min at 37 °C with 5% bovine serum albumin (BSA, Carl Roth, Karlsruhe, Germany) and washed with FACS buffer (PBS, 1% FCS, 0.02% Natrium-Azide). Samples were incubated with RSV F specific antibody (18F12) or LCMV GP-specific hybridoma supernatant (WEN4) for 1 h at room temperature. If WEN4 was used, samples were fixed with 1.5% formaldehyde for 20 min at room temperature and washed once prior to incubation with supernatant. Samples were washed once and incubated with allophycocyanin (APC)-coupled anti-mouse antibody for 30 min at room temperature in the dark. After two washing steps with FACS buffer, samples were fixed with 1.5% formaldehyde (Carl Roth, Karlsruhe, Germany) and analyzed by flow cytometry (quantification of mean fluorescence intensity).

### 2.10. Virus Capture Assay

First, 96-well Maxisorp plates (Nunc, Fisher Scientific, Vienna, Austria) were coated with 25 µg/well of polyclonal Rabbit Anti-Mouse Immunoglobulin (DAKO, Agilent, Vienna, Austria) in 0.2 M Na_2_CO_3_-NaHCO_3_, pH 9.5, overnight at 4 °C. Plates were washed three times with PBS and a second antibody was added (RSV F (18F12) (50 µg/well) or hybridoma supernatant (LCMV GP (KL25) (~25 µg/well)). Plates were incubated overnight at 4 °C and washed three times with PBS. Per well, 2 × 10^7^ TCID_50_ of VSV-GP or VSV-GP-F_syn_ were added (diluted in RPMI Medium 1640 (Gibco, Carlsbad, CA, USA)) and plates were incubated overnight at 4 °C. Plates were washed three times with RPMI Medium 1640 and captured virus was dissolved in 50 µL of NUCLISENS^®^ lysis buffer (bioMérieux Austria G.m.b.H, Vienna, Austria). RNA was isolated from lysed virus with the NUCLISENS^®^ EASYMAG^®^ System (bioMérieux Austria G.m.b.H, Vienna, Austria).

### 2.11. RSV Challenge Experiments

Female 6-8 week old BALB/c mice (Janvier) were immunized at indicated time points with VSV-GP-F_syn_, VSV-GP or formalin-inactivated RSV (FI-RSV). For intramuscular and intranasal immunization of VSV-GP vectors 10^7^ TCID_50_ were used. FI-RSV was applied at a dose of 10^6^ plaque forming units (PFU) intramuscularly. Four weeks after each immunization or 20 weeks after the boost, EDTA plasma was collected for detection of RSV neutralizing antibodies. Mice were infected with 10^6^ PFU RSV, Long Strain (ATCC VR-26), by intranasal inoculation. Five days after infection, mice were sacrificed, and lungs were removed. Lung tissue was disrupted using a tissue lyser (SpeedMill PLUS, Analytik Jena AG, Jena, Germany) and 8 beads (1 min, continuous) and centrifuged for 3 min at 15,000 rpm and 4 °C in a table top centrifuge. Supernatant was used for RNA isolation with RNeasy Mini Kit (QIAGEN, Hilden, Germany) according to manufactures recommendations. RNA concentration was measured using an Epoch Reader (BioTek Instruments, Inc., Winooski, VT, USA) and stored at −80 °C until further use.

### 2.12. qPCR

qPCR from isolated lung RNA was performed using QuantiTect SYBR^®^ Green RT-PCR Kit (QIAGEN, Hilden, Germany) with primers RSV-1 (5′-AGA TCA ACT TCT GTC ATC CAG CAA-3′) and RSV-2 (5′-GCA CAT CAT AAT TAG GAG TAT CAA T-3′). To quantify viral copies in isolated RNA, a RSV RNA-standard curve, ranging from 5 × 10^6^ to 50 copies per reaction and diluted in 1 µg polyA-RNA to mimic presence of cellular RNA, was used (as described previously [37]). VSV N qPCR was performed with 1 µL of RNA using the iTaq™ Universal Probes One-Step Kit (Bio-Rad, Hercules, CA, USA) with primers VSV N-1 (5′-AGT ACC GGA GGA TTG ACG ACT AAT-3′), VSV N-2 (5′-TCA AAC CAT CCG AGC CAT TC-3′) and FAM-labelled probe (5′-FAM-ACC GCC ACA AGG CAG AGA TGT GGT-BHQ-3′).

### 2.13. RSV Neutralization Assay

Plasma samples were incubated for 30 min at 56 °C to inactivate complement. Samples were serially 1:2 diluted in HBSS and pre-incubated with rgRSV, an RSV variant containing GFP as additional transgene, for 60 min. Subsequently, HEp2 cells were added and the plates were incubated for 48 h at 37 °C. GFP-expressing plaques were quantified by fluorescent microscopy. Plasma dilution, at which 50% of virus infection was inhibited, was defined as titer (IC_50_).

### 2.14. Statistical Analysis

Statistical analysis was performed using GraphPad prism software (GraphPad Software, Inc., La Jolla, CA, USA) as indicated in the figure legends.

## 3. Results

### 3.1. Incorporation of RSV F and LCMV GP Did Not Interfere with Each Other

In a first step, we analyzed the incorporation of different variants of the RSV fusion protein F into VSV particles. Additionally, we also wanted to determine whether the presence of LCMV GP limits the incorporation of RSV F or vice versa. We used either the wild type (F_wt_) or a codon-optimized (F_syn_) version of RSV F. Additionally, we used a chimeric protein, consisting of the extracellular part of a codon-optimized RSV F fused to the transmembrane domain and cytoplasmatic tail of VSV G (F_syn_:G), as for HIV envelope it was shown previously that such chimeric proteins are incorporated better into VSV particles [31,38].

**Figure 1:**
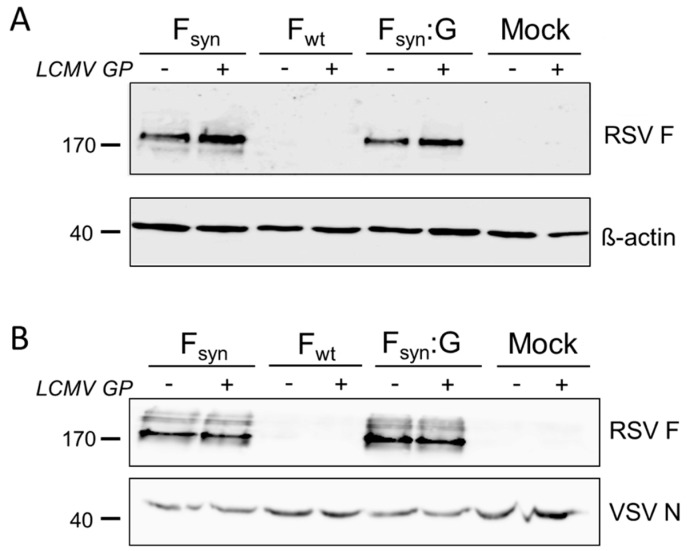
Lymphocytic choriomeningitis virus (LCMV) glycoprotein (GP) does not inhibit incorporation of respiratory syncytial virus (RSV) fusion protein (F) into vesicular stomatitis virus (VSV)-GP particles. 293T cells were transfected with expression plasmids for the wild type RSV F (F_wt_), a codon optimized variant (F_syn_) or a chimeric protein encoding the extracellular part of the codon optimized F and the transmembrane domain and the cytoplasmatic tail of VSV-G (F_syn_:G) alone or in combination with an expression plasmid for LCMV GP. As controls, cells were left untransfected or the LCMV GP plasmid was transfected alone. (**A**) Cell lysates were prepared 24 h after transfection and analyzed via Western blotting for expression of RSV F (upper blot). As loading control an antibody against β-actin was used (lower blot); (**B**) cells were infected with the single-cycle infectious VSV*ΔG virus and supernatants were collected 24 h after infection. Virus was concentrated via low speed centrifugation through a sucrose cushion. Purified virus particles were analyzed via Western blotting for incorporation of RSV F (upper blot). As loading control, the same membrane was probed with an antibody against the VSV nucleoprotein (lower blot).We next analyzed the efficiency of incorporation of RSV F protein variants into VSV-GP particles by infection of transfected cells with replication defective VSV*ΔG virus, in which the gene encoding the envelope glycoprotein G is deleted and that expresses GFP. We purified newly generated virus particles via low speed centrifugation through a sucrose cushion and checked for incorporation of RSV F by Western blot analysis. As in F_wt_ transfected cells (Figure 1A), F protein was also not detectable in VSV*ΔG particles produced on these cells (Figure 1B). In contrast, high amounts of RSV F were detected in VSV*ΔG particles produced on F_syn_ or F_syn_:G transfected cells (Figure 1B). However, fusion of F to the VSV-G transmembrane domain and cytoplasmatic tail did not increase incorporation into VSV-GP particles. The amount of VSV was normalized to the amount of N-protein on the Western blot analysis. We also found that the co-expression of LCMV GP did not limit incorporation of RSV F into viral particles, neither for F_syn_ nor for F_syn_:G (Figure 1B). To study whether incorporation of RSV F into the viral particle alters the tropism of VSV-GP, we used VSV*ΔG particles either containing only RSV F_syn_ or F_syn_ together with LCMV GP to infect the African green monkey kidney cell lines Vero, and quantified GFP^+^ cells by FACS. VSV*ΔG particles produced on LCMV GP expressing cells or produced on naïve cells (mock) were used as positive and negative controls. Vero cells were efficiently infected with particles produced on GP expressing cells. However, VSV*ΔG particles with RSV F only were not infectious (Supplementary Figure S1).

After transfection of 293T cells with expression plasmids encoding F_wt_, F_syn_ or F_syn_:G, we analyzed expression of RSV F in cell lysates. As a negative control, mock transfected cells were used. Co-transfection of a GFP-plasmid served as control for transfection levels (data not shown). To analyze interference of RSV F with LCMV GP, additional samples were co-transfected with LCMV GP. Figure 1A shows that both codon-optimized RSV F variants, F_syn_ and F_syn_:G, were expressed in similar amounts in transfected cells and that co-transfection of LCMV GP had no negative influence on protein expression. In contrast, F_wt_ was not expressed in transfected cells, neither alone nor when co-transfected with LCMV GP. This observation was in line with previously published results [39].

### 3.2. RSV F was Efficiently Incorporated into VSV-GP Particles and Did Not Interfere with Virus Replication

**Figure 2:**
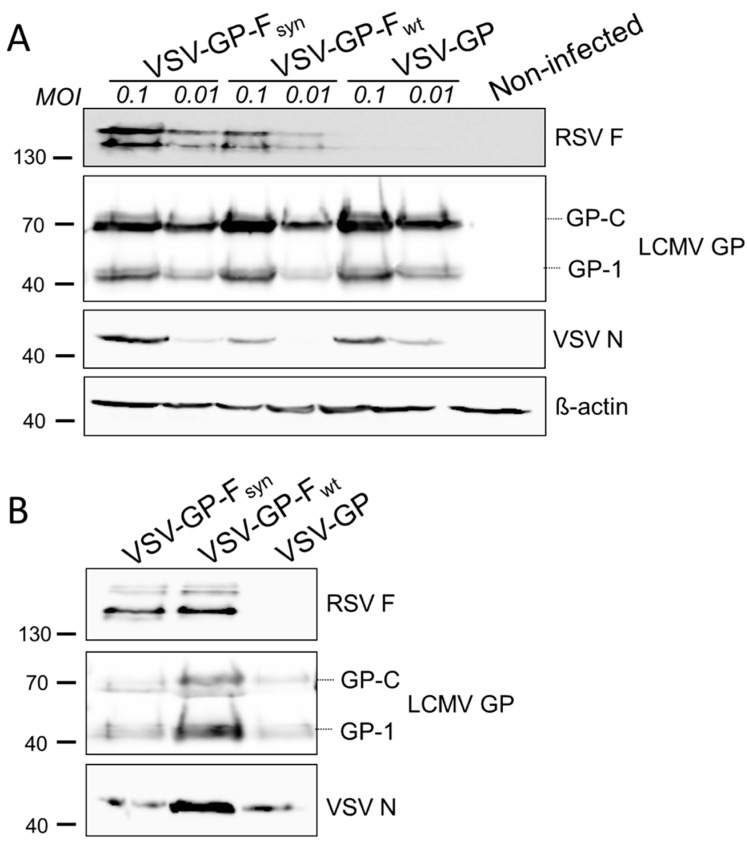
Both, wild type and codon-optimized RSV F, are efficiently expressed in VSV-GP-F infected cells and RSV F is efficiently incorporated in VSV-GP-F particles. (**A**) BHK-21 cells were infected with an multiplicity of infection (MOI) of 0.1 or 0.01 with replication competent VSV-GP variants containing a wild type (VSV-GP-F_wt_) or a codon optimized version of RSV F (VSV-GP-F_syn_). As negative control, cells were infected with VSV-GP without additional transgene. Then, 24 h after infection, cell lysates were prepared and analyzed by Western blot using RSV F-specific (top row), LCMV GP-specific (2nd row), VSV N-specific (3rd row), or actin-specific (lower row) antibodies; (**B**) virus stocks were produced on BHK-21 cells, concentrated via low speed centrifugation through a sucrose cushion and purified virus stocks of VSV-GP-F_wt_ and VSV-GP-F_syn_ were analyzed via Western blotting using RSV F-specific (top row), LCMV GP-specific (middle row), or VSV N-specific (lower row) antibodies. As negative control, VSV-GP without additional transgene was used.

To generate replication competent VSV-GP-based viral constructs, we inserted the sequence of F_wt_ or F_syn_ into the VSV-GP backbone at position 5 as an additional gene. Since both codon-optimized variants were expressed and incorporated at comparable levels we focused on F_syn_ and did not include F_syn_:G. Although we and others observed that the F_wt_ protein was not efficiently produced in transfected cells [39,40], we also introduced F_wt_ into the replication competent VSV-GP, presuming that codon-optimization may not be critical when F is transcribed by the viral polymerase in the cytoplasm. We generated infectious recombinant viruses for both variants (named VSV-GP-F_wt_ and VSV-GP-F_syn_) and in a first step, analyzed the expression of both RSV F variants upon infection of BHK-21 cells (Figure 2A). Cells were infected with an MOI of 0.1 or 0.01 of VSV-GP-F_wt_, VSV-GP-F_syn_ or, as a control, VSV-GP, and cell lysates were prepared 24 h after infection. Figure 2A shows that both RSV F variants were efficiently produced by infected cells in comparable amounts. To control for the level of virus replication we used the VSV N protein band on the Western blot. At the lower MOI (0.01), however, VSV-N protein was hardly detectable after 24 h. Likewise, F expression was also lower for MOI 0.01 than 0.1 after 24 h. When analyzing purified virus stocks via Western blot analysis, we clearly observed an incorporation of both RSV F variants into VSV-GP particles, which did not affect LCMV GP incorporation (Figure 2B).

**Figure 3:**
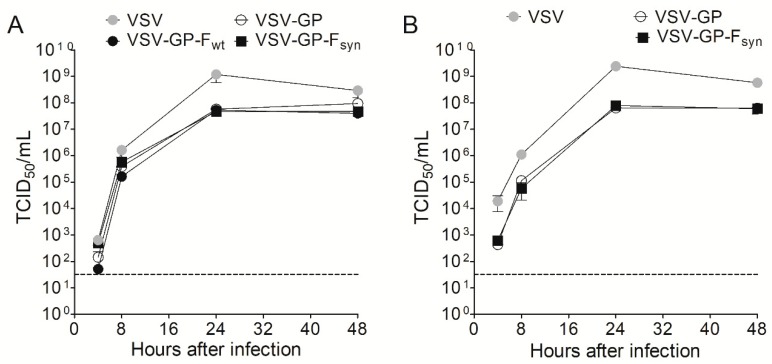
RSV F does not attenuate VSV-GP. (**A**) BHK-21 or (**B**) Vero cells were infected with an MOI of 0.1 with VSV wild type, VSV-GP and VSV-GP variants containing a wild type or a codon optimized version of RSV F (VSV-GP-F_wt_ and VSV-GP-F_syn_, respectively) (*n* = 3). At indicated time points, the supernatant was collected and analyzed for viral titer via tissue culture infectious dose 50% (TCID_50_) assay. Shown are mean ± SEM. The dotted line indicates the detection limit of the assay.

We also confirmed incorporation of RSV F into the viral particle by flow virometry analysis [41] and found that both F variants were incorporated at similar levels (Supplementary Figure S2A,B). To exclude that the F signal we observed in the Western blot analysis and FACS staining was derived from cell debris rather than from VSV-GP particles we performed a virus capture assay and determined viral genome copies of viruses captured with an LCMV GP- or an RSV F-specific antibody. While both viruses, VSV-GP and VSV-GP-F, could be captured with a LCMV GP-specific antibody, only VSV-GP-F was captured using an F-specific antibody (Supplementary Figure S2C). To further characterize VSV-GP-F_wt_ and VSV-GP-F_syn_, we compared the replication kinetics of these two viruses to VSV-GP and VSV wild-type. For this, we infected BHK-21 cells with an MOI of 0.1 of the corresponding viruses and determined the titer in the supernatant 4, 8, 24 and 48 h after infection by TCID_50_. As we described previously for virus variants containing the model antigen ovalbumin [30], we also measured a slight reduction of viral growth of ~1 log for VSV-GP, as compared to wild type VSV. However, replication and viral titer were not further impaired by including F_wt_ or F_syn_ into VSV-GP (Figure 3A). For RSV particles it has been shown that on some cell lines, as for example Vero cells, F alone is sufficient for replication and therefore a ΔG RSV variant is infectious [15]. To further analyze if F affects VSV-GP replication on such cells, we repeated the replication kinetics on Vero cells. However, we found no differences in replication of VSV-GP variants with or without F, indicating again that F does not contribute to VSV-GP-F’s tropism (Figure 3B).

### 3.3. Immunization with VSV-GP-F_syn_ Protected Mice from RSV Infection

**Figure 4:**
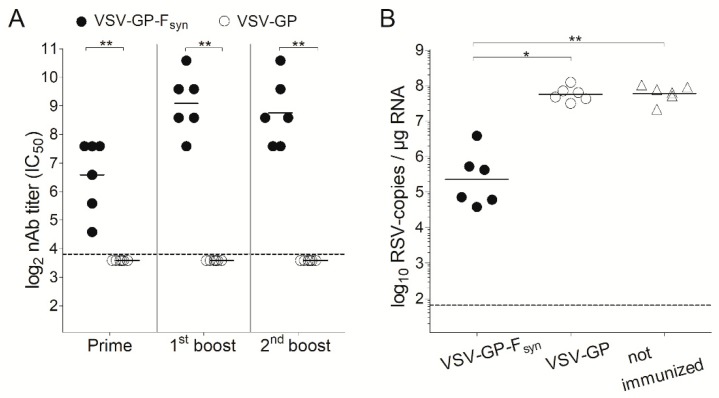
VSV-GP-F_syn_ vaccinated mice are protected against RSV challenge. BALB/c mice (*n* = 6) were immunized three times intramuscularly (weeks 0, 4 and 8) with 10^7^ TCID_50_ of VSV-GP or a VSV-GP variant containing a codon optimized version of RSV F (VSV-GP-F_syn_). Control mice were left unimmunized. (**A**) Four weeks after each immunization, ethylenediaminetetraacetic acid (EDTA) plasma was collected and analyzed for the titer of RSV neutralizing antibodies. Statistical significances were determined by nonparametric statistics (two-tailed, Mann–Whitney test) (*, *p* ≤ 0.05; **, *p* ≤ 0.01); (**B**) eight weeks after the last immunization, mice were infected with 10^6^ plaque forming units (PFU) of replication competent RSV. On day 5 after infection, mice were sacrificed, and lungs were analyzed for the titer of RSV by qPCR. Statistical significances were determined in a one-way analysis of variance (Kruskal–Wallis test) followed by Dunn’s Multiple Comparison Test (*, *p* ≤ 0.05; **, *p* ≤ 0.01). (**A**,**B**) Geometric mean is indicated; the dotted lines indicate the detection limits of the assays.

To analyze immunogenicity of the VSV-GP vaccine vector, we determined whether VSV-GP-F induced protection in an RSV mouse challenge model. In our *in vitro* studies, we had not observed a difference between VSV-GP-F_syn_ and VSV-GP-F_wt_ with regard to protein expression, incorporation of F into viral particles and replication kinetics. However, former studies have shown an advantage of using a codon-optimized F in terms of expression levels and consequently immunogenicity [34]. Therefore, we chose VSV-GP-F_syn_ for *in vivo* experiments. In a first experiment, we immunized BALB/c mice three times intramuscularly with VSV-GP-F_syn_ in four-week intervals. As controls, we included VSV-GP without an additional transgene and mice, which were not immunized. Four weeks after each immunization, we collected plasma and quantified RSV nAb titers. As expected, we could not detect RSV nAbs in the plasma of any of the VSV-GP immunized animals. In contrast, in all of the VSV-GP-F_syn_ immunized animals, RSV nAbs were induced already after the first immunization. RSV nAbs were boosted once after the second immunization but did not increase further after the third immunization (Figure 4A). Eight weeks after the third immunization, the animals were infected intranasally with RSV. Challenge of mice with RSV induced weight loss in all groups, independent of the immunization and no signs of an immunization-induced enhanced disease were observed (Supplementary Figure S3). Viral load after infection of BALB/c mice with RSV peaks around day 5 after infection [34,42], therefore we isolated lungs and quantified viral load at this time point by quantitative PCR (qPCR). We measured high numbers of RSV copies in non-immunized or VSV-GP control vector immunized mice. In contrast, VSV-GP-F_syn_ immunized animals were significantly protected from RSV infection and showed ~2.5 log reduction in RSV copies in the lungs relative to both control groups (Figure 4B).

**Figure 5:**
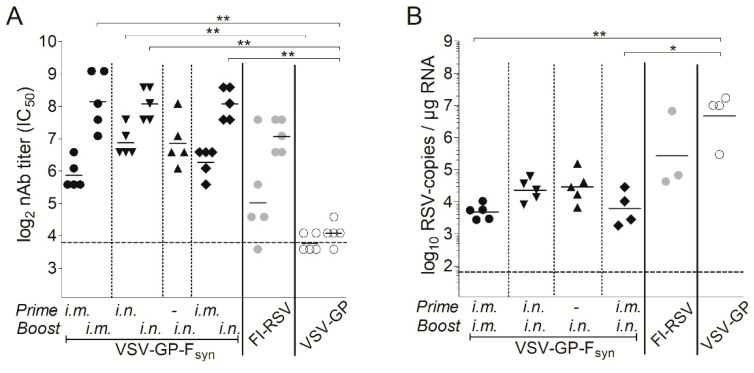
A single intranasal immunization with VSV-GP-F_syn_ induces RSV-neutralizing antibodies and reduces RSV viral load by 2 log. BALB/c mice (*n* = 5) were immunized in weeks 0 and 4 intramuscularly with 10^7^ TCID_50_ VSV-GP-F_syn_, VSV-GP or 10^6^ formalin inactivated (FI)-RSV, intranasal with 10^7^ VSV-GP-F_syn_ or first intramuscular followed by intranasal with 10^7^ VSV-GP-F_syn_. One group received a single intranasal immunization with 10^7^ VSV-GP-F_syn_ in week 4. (**A**) EDTA plasma was collected 4 weeks after prime and boost and analyzed for the titer of RSV neutralizing antibodies; (**B**) in week 4 after boost, mice were infected with 10^6^ PFU of replication competent RSV. On day 5 after infection, mice were sacrificed, and lungs were analyzed for the titer of RSV by qPCR. Statistical significances were determined in a one-way analysis of variance (Kruskal–Wallis test) followed by Dunn’s Multiple Comparison Test (*, *p* ≤ 0.05; **, *p* ≤ 0.01). Geometric mean is indicated; the dotted lines indicate the detection limits of the assays.

**Figure 6:**
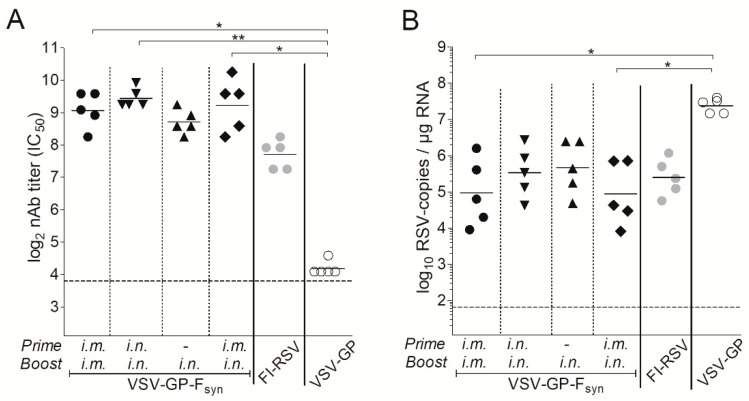
RSV-neutralizing antibodies induced by immunization with VSV-GP-F_syn_ are long-lasting. BALB/c mice (*n* = 5) were immunized in weeks 0 and 4 intramuscularly with 10^7^ TCID_50_ VSV-GP-F_syn_, VSV-GP or 10^6^ FI-RSV, intranasally with 10^7^ VSV-GP-F_syn_ or first intramuscularly followed by intranasally with 10^7^ VSV-GP-F_syn_. One group received a single intranasal immunization with 10^7^ VSV-GP-F_syn_ in week 4. (**A**) EDTA plasma was collected 20 weeks after boost and analyzed for the titer of RSV neutralizing antibodies; (**B**) in week 20 after boost, mice were infected with 10^6^ PFU of replication competent RSV. On day 5 after infection, mice were sacrificed, and lungs were analyzed for the titer of RSV by qPCR. Statistical significances were determined in a one-way analysis of variance (Kruskal–Wallis test) followed by Dunn’s Multiple Comparison Test (*, *p* ≤ 0.05; **, *p* ≤ 0.01). Geometric mean is indicated; the dotted lines indicate the detection limits of the assays.

In a next experiment, we aimed to determine the impact of the immunization route and if a single immunization would also be protective. BALB/c mice were immunized intramuscularly or intranasally with VSV-GP-F_syn_ by homologous prime boost vaccination regimen, or only once intranasally. Additionally, mice which received an intramuscular prime and an intranasal boost were included. As a positive control, we included animals vaccinated with formalin inactivated RSV (FI-RSV). Animals immunized with VSV-GP lacked RSV nAbs (Figure 5A). In all VSV-GP-F_syn_-immunized animals, RSV nAbs were induced after prime and could be efficiently boosted (Figure 5A). Mice, which received a single intranasal immunization with VSV-GP-F_syn_ had slightly lower titers (comparable to FI-RSV group). The viral load in all groups was decreased compared to mice receiving FI-RSV or VSV-GP (Figure 5B). It should be noted that single or multiple intranasal immunizations were similarly protective after challenge (Figure 5B). Lastly, we aimed to determine durability of RSV nAbs induced by VSV-GP-F immunization. Therefore, RSV nAbs were determined in an additional group of mice 20 weeks after the last immunization. Titers of RSV nAbs after 20 weeks were still high (Figure 6A) and in similar range as 4 weeks after the boost (Figure 5A). These long-lasting antibodies translated also in a long-lasting protection. When mice were challenged 20 weeks after the last immunization, viral load was reduced by ~2–3-logs compared to control mice (Figure 6B). The difference between the control and immunized groups was less pronounced than for animals challenged after 4 weeks (Figure 5B), but in the groups receiving intramuscular injections, reduction in viral load was still significant.

## 4. Discussion

In a previous study, using ovalbumin as model antigen, we showed that VSV-GP is a new promising candidate vaccine vector [30]. Vaccination with VSV-GP protected mice from an infection with *Listeria*, expressing ovalbumin, as model organism. Control of *Listeria* is mainly mediated by antigen-specific CTLs which were efficiently induced by VSV-GP. Here, we now explored VSV-GP as vaccine vector against RSV, where antibodies play a more important role. We included RSV F into the VSV-GP vector with the aim to induce RSV neutralizing antibodies.

It is interesting that in contrast to plasmid transfection, RSV F did not need to be codon optimized to be efficiently expressed from the VSV-GP genome. Low cytoplasmic mRNA level and a premature polyadenylation site are the reason for the lack of F expression from plasmid DNA [39]. A likely explanation that codon-optimization is not necessary for the VSV-encoded transgene is an efficient transcription of these by the viral polymerase and the expression in the cytoplasm, thereby circumventing nuclear mRNA export. In contrast to our results, Liang and colleagues found that for a live attenuated chimeric bovine/human parainfluenza virus vector a codon-optimized version of the pre-fusion F was better expressed than the non-codon optimized [43]. The expression of codon optimized sequences is proposed to be dependent on of the availability of tRNA molecules in the cell. Therefore, the expression is enhanced in mammalian cells using mammalian codon usage. The enhanced expression of codon-optimized F also translated into a better antibody response in a hamster model. As we used a different algorithm for codon-optimization compared to the ideal construct from Liang *et al.*, although in both cases the constructs were optimized for human codon usage, it might be worth to further optimize the F insert in the VSV-GP vector, which could then potentially even enhance nAb responses achieved by VSV-GP-F.

A strong advantage of VSV-based vectors is that they can incorporate foreign antigens into their envelope. We recently showed that for HIV incorporation of HIV Env into the VSV-GP particle indeed enhanced antibody titers in mice relative to Env constructs that were primarily expressed on the surface of infected cells [31]. Additionally, RSV F incorporation into parainfluenza virus vector particles improved nAb response to RSV F [44]. Therefore, in a first step, we confirmed that RSV F was efficiently incorporated into VSV-GP particles using Western blot and flow virometry analysis. RSV F incorporation was not hindered by LCMV GP and did not attenuate the virus. Our results are in line with a study by Kahn and colleagues who showed that RSV F and also RSV G were efficiently incorporated into wild-type VSV particles [45]. Although for other viral glycoproteins such as HIV Env it has been shown that incorporation into VSV-based vectors could be enhanced by generating Env/VSV-G chimeric fusion proteins [38], we found that this was not the case for incorporation of RSV F into VSV-GP. An explanation for this might be that HIV and VSV bud from different regions of the plasma membrane and therefore also HIV Env and VSV G are transported to different sites [46]. Redirecting HIV Env to VSV budding sites by the VSV G cytoplasmatic tail therefore enhanced levels of the chimeric Env in VSV particles. This might not be necessary in the case of RSV F.

In a next step, we analyzed if RSV F can mediate infectivity of VSV particles. This is especially important as an infectious F incorporated into the VSV-GP particle might change the tropism of VSV-GP-F particles compared to the parental VSV-GP and therefore might also change the safety profile of the vector. For VSV-GP we previously showed that the virus is safe in mice at high doses and is completely non-toxic upon systemic injection in immunocompetent and immunodeficient mice [33,47]. Even high doses of intracranial injection were well tolerated [32]. In pre-clinical models, the rVSV-ZEBOV vaccine, a VSV-based Ebola virus vaccine, had a safety profile comparable to VSV-GP and was recently tested in several clinical trials, including a phase I trial in 6–12-year-old children. The vaccine was found to be safe and well tolerated also in the children [48].

To analyze whether infectivity was mediated by F, we produced replication defective VSV*ΔG particles expressing GFP either on cells expressing only F or F and LCMV GP in combination. This resulted in VSV particles with either F as sole glycoprotein in the viral envelope or F and GP together. Only particles containing GP were infectious whereas F alone was not sufficient to infect cells. Our findings are in contrast to an earlier study by Kahn and colleagues who found that F mediated infectivity of VSV particles [45]. However, they used particles with F and VSV G on the surface and blocked VSV G infectivity by increasing the endosomal pH using ammonium chloride. In this setting, attachment might be still mediated via VSV G, leaving only the fusion function for F as also in the natural context during RSV infection. An RSV variant with a deleted G was shown to be infectious, but this was strongly dependent on the target cell line and *in vivo* infectivity is strongly reduced for this ΔG RSV variant [15]. Although we used Vero cells, one of the cell lines for which Teng and colleagues showed that it is susceptible for the ΔG RSV variant, we did not see any infectivity of VSV*ΔG particles containing F as sole glycoprotein. Therefore, we can assume that tropism and consequently safety of VSV-GP-F should not be altered compared to the parental VSV-GP.

Presentation of RSV F in a natural conformation and maybe as flexible surface protein that can change between pre- and post-fusion state might be especially important, as previous studies with a formalin-inactivated RSV vaccine resulted in an enhanced disease and even the death of two vaccinated children [20]. It is discussed that the induction of non-neutralizing, binding or only low affinity neutralizing antibodies is one of the reasons for this enhanced infection [28]. Induction of these kinds of antibodies might be avoided by using F in a native-like conformation. We hypothesize that F folding is more native-like on the surface of an infectious virus compared to F on the surface of formalin-treated virus and might therefore also induce better RSV nAbs. Indeed, we found higher titers of RSV nAbs in the plasma of VSV-GP-F immunized than FI-RSV immunized mice. Alike others [20,29,49], we measured nAbs after immunization with FI-RSV and slightly decreased viral titers in the lung, but animals were not protected as efficiently as VSV-GP-F_syn_ immunized animals.

VSV-based vectors have already been previously tested in pre-clinical models as RSV vaccine and mediated protection against RSV infection in mice [50,51]. However, all vectors containing VSV G have the problem of potential neurotoxicity. This is especially true for intranasal application in young mice, where the virus needs massive attenuations to be safe [52]. However, small children are one of the major target groups for an RSV vaccine. A potentially safe alternative are VSVΔG variants, where the glycoprotein of VSV is deleted. However, these viruses are difficult to produce in large scale under GMP conditions as the virus needs to be amplified on cells expressing VSV G. As VSV G is fusogenic and toxic it is difficult to make cell lines stably expressing VSV G. VSV-GP should circumvent these disadvantages of other VSV-based RSV vaccine candidates as it completely lacks VSV’s neurotoxicity and on the other hand is fully replication-competent and easy to produce [30,32].

The titer of RSV-specific (neutralizing) antibodies is the ‘correlate of protection’ (CoP), while the immune response arising from CD4^+^, CD8^+^ and innate cells are regarded as ‘co-CoPs’ [7]. Serum nAb titers are indicators for the risk of RSV-associated hospitalization: nAb titers of ≥6 or ≥8 were found to be 3.5 and 2.9 times (for RSV-A and B, respectively) more likely of preventing hospitalization [8]. As this refers to the human system, these values cannot be directly compared to the murine system; however, nAb titers induced by VSV-GP-F are in the upper range compared to other studies [29,43,53]. Although we were not able to completely protect from RSV challenge, we could, already with a single immunization, reduce viral load significantly. Reduction in viral load is an important achievement, as severity of disease correlates with viral load [54,55,56]. In contrast to an adenoviral vector, we did not observe a better protection for intranasal immunization compared to intramuscular which might be due to the different tropism of adenoviral and VSV-GP vectors [37]. However, even a single intranasal immunization induced mean RSV nAb titer of 6.85 log2 and reduced mean RSV load after challenge by more than two logs.

Our results provide first efficacy data on a novel viral vector vaccine platform against RSV thereby underlining the potency of VSV-GP as a vaccine vector. The vaccine potency together with VSV-GP’s favorable safety profile, the ability of the vector to incorporate F into the viral envelope allowing a native-like presentation of F and the possibility for intranasal application make VSV-GP an interesting candidate for RSV vaccine development.

## Supplementary Materials

The following are available online at https://www.mdpi.com/2076-393X/7/3/59/s1, Figure S1: VSV particles with RSV F as only glycoprotein are not infectious, Figure S2: RSV F is incorporated in viral particles, Figure S3: Weight loss after challenge with RSV
