# Supplementary material for: RSV Vaccine Based on Rhabdoviral Vector Protects after Single Immunization
Source: Vaccines (Basel). 2019 Jul 3;7(3):59. doi: 10.3390/vaccines7030059 (PMC6790003; doi:10.3390/vaccines7030059)
Supplement: Supplementary file 1 [file vaccines-07-00059-s001.pdf]

Supplementary material

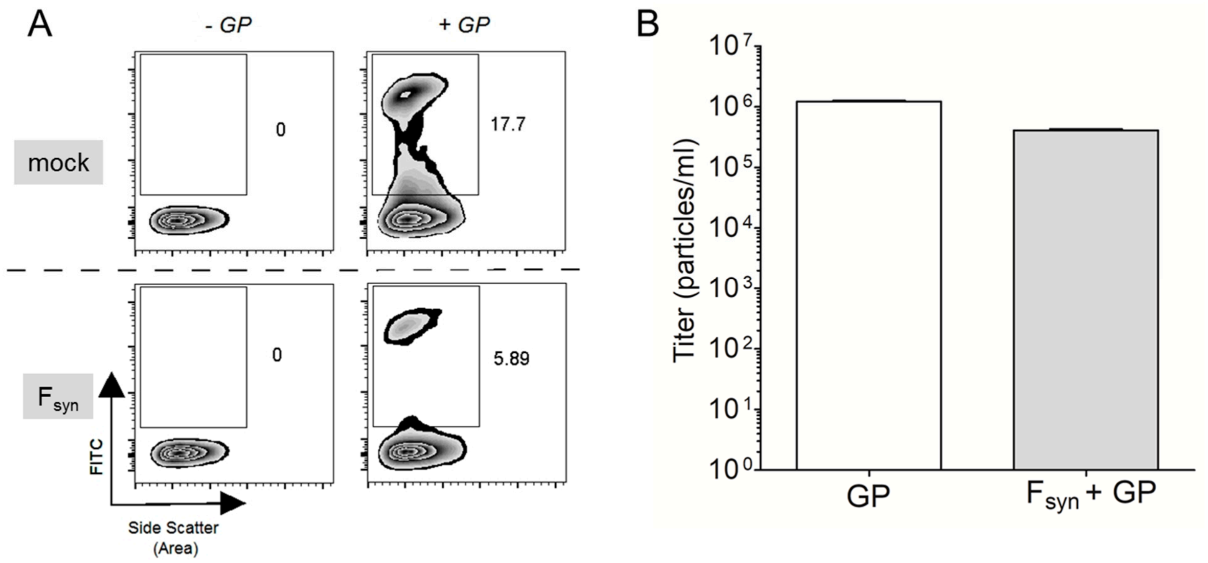

Figure S1. VSV particles with RSV F as only glycoprotein are not infectious. 293T cells were transfected with expression plasmids for a codon optimized variant of RSV F (F_{syn}) alone or in combination with an expression plasmid for LCMV GP. As controls, cells were left untransfected (mock) or the LCMV GP plasmid was transfected alone. Cells were infected with the single-cycle infectious VSV*ΔG virus (expressing GFP) and VSV*ΔG particles pseudotyped with LCMV GP and/or RSV F were collected after 24 hours. Pseudotyped VSV*ΔG viruses were incubated with serum containing VSV-G neutralizing antibodies and subsequently titrated on Vero cells by flow cytometric quantification of GFP^+ cells (n = 3). (A) Representative scatter dot plots. Indicated are percentages of gated GFP^+ cells. (B) Calculated titer of pseudotyped VSV*ΔG viruses. Shown are mean ± SEM.

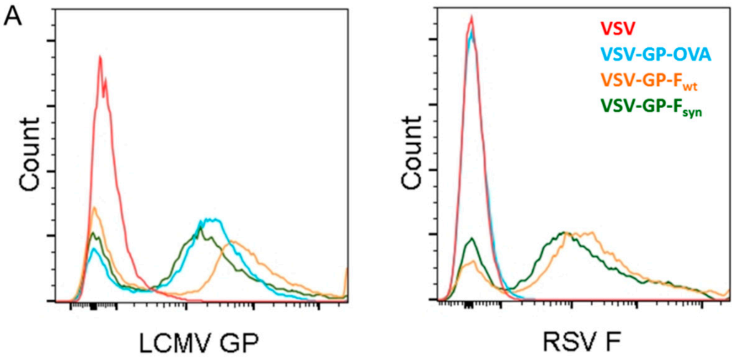

B

	LCMV GP (±SEM)	RSV F (±SEM)	Ratio
VSV-GP-F_{wt}	14575 (±427)	11719 (±441)	0.80
VSV-GP-F_{syn}	8929 (±791)	6668 (±340)	0.75

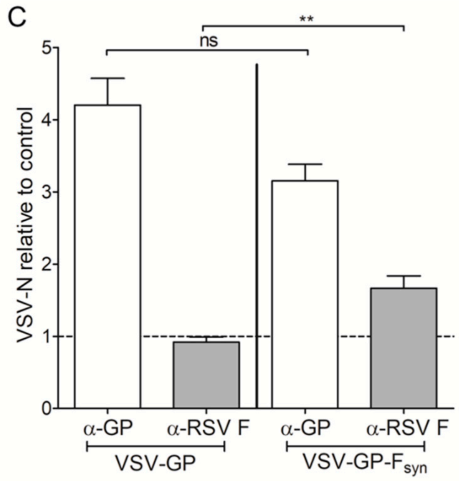

Figure S2. RSV F is incorporated in viral particles. Purified virus stocks were analyzed by flow virometry for incorporation of LCMV GP (WEN4) (left) and RSV F (18F12) (right) into the viral particle (n≥2). Mean fluorescence intensities (MFI) were quantified. (A) Representative histograms (red: VSV; blue: VSV-GP-OVA; orange: VSV-GP-F_{wt}; green: VSV-GP-F_{syn}) and (B) mean MFIs with calculated ratio (the amount of RSV F in relation to LCMV GP). (C) VSV-GP or VSV-GP-F_{syn} were captured on plates coated with α-GP or α-RSV-F antibodies or non-coated control wells (n = 4 per virus and antibody). RNA was isolated and viral genome number was determined via qPCR using VSV-N-specific primers. Control wells were set to 1 (dotted line). Shown are mean ± SEM. Statistics were determined using an unpaired t test, **$p<0.01$, ns = not significant).

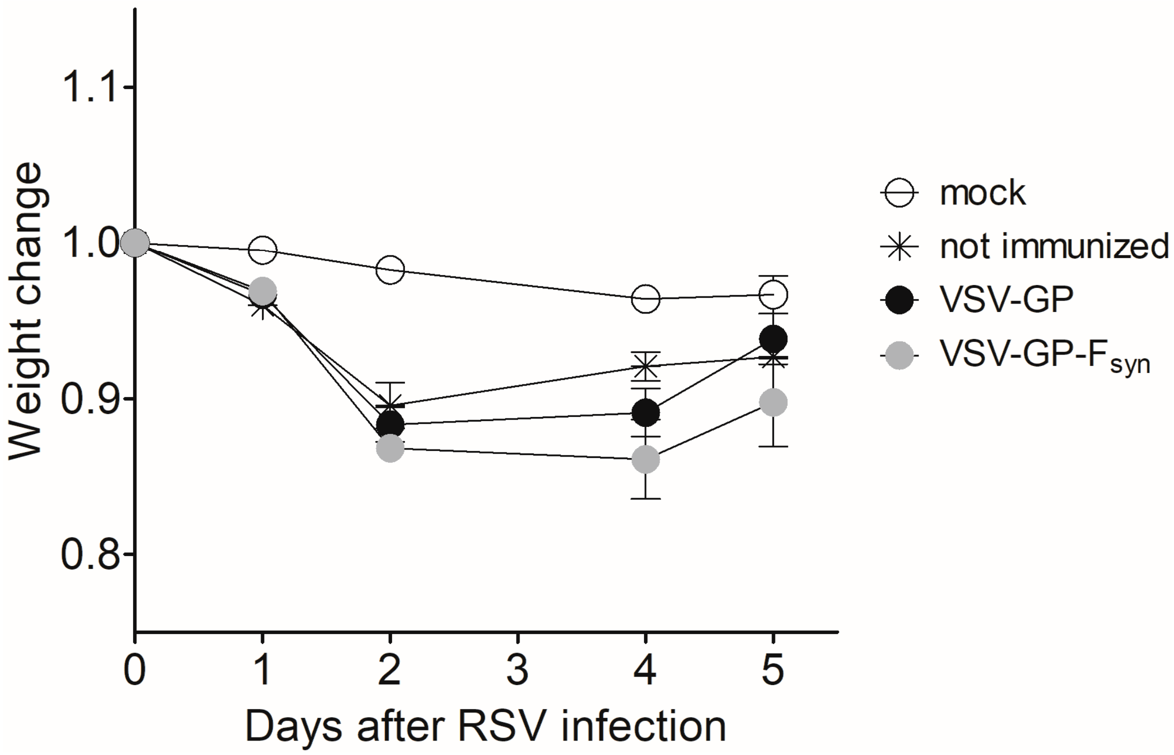

Figure S3. Weight loss after challenge with RSV. BALB/c mice (n = 6) were immunized intramuscularly with VSV-GP or a VSV-GP variant containing a codon optimized version of RSV F (VSV-GP-F_{syn}) in weeks 0, 4 and 8. Eight weeks after the last boost, mice were infected with 10^6 PFU of replication competent RSV. Mice were weighted at different days after challenge. Shown are mean ± SEM.
